# Effects of inter-twin vascular anastomoses of monochorionic twins with selective intrauterine growth restriction on the contents of placental mitochondria DNA

**DOI:** 10.1186/s12884-018-1702-8

**Published:** 2018-03-24

**Authors:** Yao-Lung Chang, An-Shine Chao, Hsiu-Huei Peng, Shuenn-Dyh Chang, Sheng-Yuan Su, Kuan-Ju Chen, Tzu-Hao Wang

**Affiliations:** 1 Department of Obstetrics and Gynecology, Chang Gung Memorial Hospital, Linkou, College of Medicine, Chang Gung University, No 5, Fu-Shin Road, Gwei-shan, Taoyuan, Taiwan; 2 School of Traditional Chinese Medicine, College of Medicine, Chang Gung University, Taoyuan, Taiwan; 3 Genomic Medicine Research Core Laboratory (GMRCL), Chang Gung Memorial Hospital, Tao-Yuan, 333 Taiwan

**Keywords:** Placenta mitochondria, Monochorionic twins, Twin-twin transfusion syndrome

## Abstract

**Background:**

Placental mitochondrial DNA (mtDNA) has been proposed to be an indicator for placental hypoxia. This study was designed to evaluate the effect of vascular anastomoses between monochorionic (MC) twins on placental mtDNA.

**Methods:**

In this study, twin-twin transfusion syndrome (TTTS) treated with laser therapy and MC twins without TTTS (without laser therapy) resulting in two live babies were included in this study. The placental mtDNA fold changes (FC) between the small and large twins were analyzed using real-time quantitative PCR. TTTS twins with selective intrauterine growth restriction (sIUGR) are categorized as group 1, TTTS without sIUGR as group 2, MC twins without TTTS but with sIUGR as group 3, and MC twins without both TTTS and sIUGR as group 4.

**Results:**

There were seven cases in group 1, eight in group 2, 26 in group 3, and 24 in group 4 cases. The placental mtDNA FC were significantly higher in group 1 (1.57 ± 0.9) compared to that of the group 3 (0.86 ± 0.6).

**Conclusion:**

In MC twin pregnancies with sIUGR, the placental mtDNA FC between the small and large twins are different between cases with and without inter-twin anastomoses. These findings suggest that the inter-twin anastomoses in the MC twins with sIUGR may provide rescue perfusion from the appropriate-for-gestational-age twin to the sIUGR one.

**Electronic supplementary material:**

The online version of this article (10.1186/s12884-018-1702-8) contains supplementary material, which is available to authorized users.

## Background

Every mitochondrion contains 2–10 copies of mitochondrial DNA (mtDNA) in its matrix, resulting in more than 10,000 copies of mtDNA in each human cell [1–3]. MtDNA typically replicates during every cell cycle; each daughter cell usually maintains a relatively consistent number of copies of mtDNA [4]. However, replication of mitochondrial DNA may also be independently affected by genetic disorders [5] or environmental factors, such as oxidative stress [6, 7].

MtDNA has been shown to be increased in placental tissues of pregnancies with intrauterine growth restriction (IUGR) [8, 9]. Although the exact mechanism how the placental mtDNA increase in the case of sIUGR remains unclear, placental mtDNA contents were correlated with low oxygen levels [8]. Furthermore, the copy numbers of mitochondrial DNA were increased by changes affecting fetal growth [10]. Monochorionic (MC) twins, who share identical genetic materials, enable us to study the mechanisms of mtDNA increase in cases of IUGR without confounding effects from genetic factors.

There are three types of intertwin anastomoses in monochorionic twins: artery-to-artery (AA), vein-to-vein (VV), and artery-to-vein (AV) anastomoses. AA and VV anastomoses form direct communications on the surface of the chorionic plate and are bidirectional. Unidirectional artery-to-vein (AV) anastomoses are located deep in the placental tissue. An imbalance in intertwin transfusion through vascular anastomoses is the underlying mechanism of TTTS [11]. An effective treatment of twin-twin transfusion syndrome (TTTS) is to disrupt the vascular anastomoses in the placenta of MC twins with laser photocoagulation therapy [12, 13]. However, about 41.4% of TTTS suffer from selective FGR (sIUGR) even after successful laser therapy resulting in two live twins [14].

The development of sIUGR in MC twins is likely due to unequal placental sharing [15]. We have previously found that mitochondrial activation in cord blood and the amniocytes of the sIUGR fetus in MC twins was likely to be regulated by locally adverse placental conditions resulting in reduced blood flow, rather than genetic factors [16].

In the current study to further test the hypothesis that the twin-twin vascular anastomoses may provide a rescue perfusion to the sIUGR twin, we attempted to compare the mtDNA quantity in the placental territories of each fetus in MC twin pregnancies with or without sIUGR. Furthermore, we studied the changes in placental mtDNA contents after laser therapy for the treatment of TTTS.

## Methods

### Clinical subjects

In this study, we only analyzed the women whom ultimately gave birth at Chang Gung Memorial Hospital, Taoyuan, Taiwan, which is a tertiary referral center with 3668 beds. All women included into this study were Taiwanese. All of the MC twin gestations with TTTS during the period of this study were treated with laser therapy, and all of these pregnancies were delivered by Cesarean section.

The inclusion criteria of this study included: TTTS twins successfully treated by laser therapy resulting in two live infants with placentas that were intact enough to be studied after birth. Other MC twins without TTTS, with or without sIUGR, were also enrolled in the control group. Successful laser therapy for TTTS was defined as the resolution of polyhydramnios-oligohydramnios after the procedure, delivery of two viable babies, and no obvious residual superficial anastomoses detected via a thorough gross placental examination at delivery. Monoamniotic twin are excluded from this study.

A twin pregnancy with sIUGR was defined as an estimated fetal weight below the 10th percentile for gestational age in one twin according to a singleton growth chart, whereas the appropriate-for-gestational-age (AGA) twin was the co-twin without FGR [17]. The diagnosis of TTTS was based on the ultrasound findings defined by Quintero et al. [12]. The laser therapy were performed between TTTS diagnosed at gestational 16~ 26 weeks. In TTTS twins or MC twins without TTTS and without sIUGR (group 4), the larger twin is the one with heavier birth weight. The discordance between the twins’ weights was defined as (birth weight of the larger twin – birth weight of the smaller twin) × 100% / (birth weight of the larger twin). The discordance of placental shares was calculated similarly (see below for further details).

### Inspection and collection of placental tissues

The placentas were collected immediately after delivery. After blood was drained from the umbilical vessels, the placenta was washed with ice-cold PBS to remove all blood clots and examined for integrity of the cotyledons and membranes. The placenta was weighed after the umbilical cord and membranes were removed. In cases without TTTS, the vascular equator was defined as a border drawn in the middle of the vascular zone on the chorionic fetal surface where the communicating vessels met. In cases with TTTS, the vascular equator was defined as the area where laser therapy had been applied to coagulate anastomoses. If residual anastomosis was detected by visual inspection, the placenta was excluded from this study. The placentas were cut along the vascular equator. Each placental portion was weighed separately to record the individual placenta mass (IPM) [18]. The discordance of placental shares was calculated as (IPM of the larger twin – IPM of the smaller twin) × 100% / (IPM of the larger twin).

We collected two to three biopsies of fresh placental tissue (0.5X 0.5 X 0.5 cm) from each placental territory of each MC twin. The placental tissue biopsies were taken at the midway point between the vascular equator to its corresponding cord insertion, and at the middle layer of placenta between the maternal and fetal surfaces. Regions with obvious calcification or infarction were avoided. The placenta specimens were briefly rinsed with ice-cold PBS to remove blood, snap frozen in liquid nitrogen, and stored in a − 70 °C freezer.

Real-time quantitative polymerase chain reaction (PCR) analysis.

The protocols of mtDNA real time quantitative PCR using TaqMan PCR Core Reagent Kit and the Sequence Detection system (ABI Prism 7900, Applied Biosystems) have been previously reported [5]. Briefly, the ABI mitochondrial gene 7S encoding D-loop was used as the target gene (Hs02596861_s1, Applied Biosystems, CA, USA), and the amount of glyceraldehyde-3-phosphate dehydrogenese (GAPDH) gene was used to represent nuclear DNA concentrations. Thermal cycling was initiated with 2-min incubation at 50 °C and followed by the first denaturation period of 10 min at 95 °C, followed by 40 cycles of 95 °C for 15 s and 60 °C for 1 minute. In our laboratory, we routinely do RT-QPCR of each sample in duplicate and keep the coefficient of variation (CV) less than 15%. Whenever the CV of two values appears greater than 15% (it hardly happens), we discard that measurement and re-assay the sample. The comparative threshold cycle (Ct) method was used to analyze data [19]. The △Ct values from each sample were obtained by subtracting the value of endogenous control gene. The 2^-△△Ct^ was used to calculate relative amounts of mtDNA, represented as the placental mtDNA fold changes, between the two fetuses in MC twins.

### Data analysis

The placental mitochondrial DNA (mtDNA) fold change was defined as (placental mtDNA content of the sIUGR or smaller fetus) / (placental mtDNA content of the AGA or larger fetus). The placental mtDNA fold change in each pair of MC twins was calculated, then those data were analyzed with the SPSS software, generating means and standard deviation (S.D.) in each one of the four groups of subjects.

Statistical analysis was conducted with the SPSS software (version 11.0 for Window; SPSS Inc, Chicago, IL). Continuous variables were first tested for normal distribution using the Kolmogorov-Smirnov test. Two-sample Student *t* test or Mann-Whitney *U* test were used to compare the values of two groups, binomial factor were compared by Chi-Square test and one-way ANOVA test or Kruskal-Wallis Test (if the *p* value of Test of Homogeneity of Variances was< 0.05) were used for data more than or equal to three groups. A probability value of less than 0.05 was considered to be statistically significant.

## Results

From July 2010 to June 2014, 65 pairs of MC twins were included in this study, including 15 pairs with TTTS and 50 without TTTS (named as No TTTS). The mean gestational age of laser therapy for TTTS was 20 ± 2.7 weeks of gestation. In the group of TTTS (group 1 and 2); there were two cases as Quintero stage I, six as stage II, six as stage III, and one as stage IV, resulting in 7 pairs with sIUGR (group 1) and 8 pairs without (group 2). In the group of MC twins without TTTS, 26 cases were found to have sIUGR (group 3) and 24 cases did not develop sIUGR (group 4). The percentage of sIUGR in MC twins at our hospital, a tertiary referral center, was 52% (26/50). There were no statistical differences in maternal age at delivery, gestational age at delivery, birth weight of the smaller twin, birth weight of the larger twin, or birth weight discordance between TTTS and No TTTS twins, in cases with sIUGR (group 1 vs. group 3, Table 1, *P*1) or in the cases without sIUGR (group 2 vs. group 4, Table 1, *P*2). Because pregnancies with sIUGR were generally delivered earlier, the fetal birth weight of groups 1 and 3 were lighter than that of groups 2 and 4 (groups 1 + 3 vs. groups 2 + 4 Table 1, *P*3). Birth weight discordance was significantly higher in twins with sIUGR than those without (Table 1, *P*3).

**Table 1:**
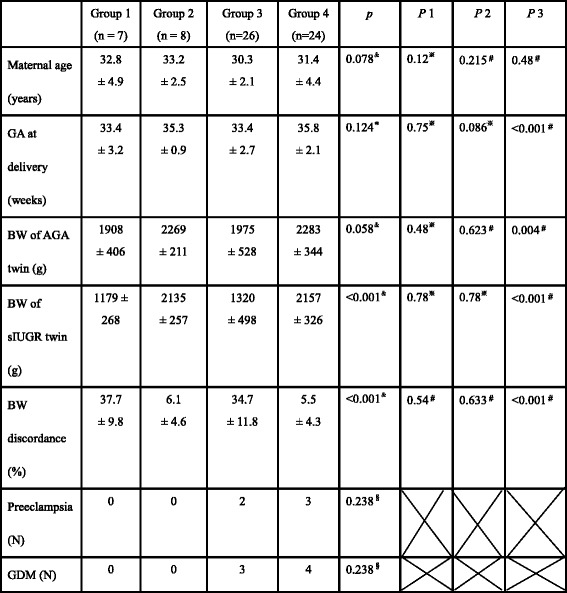
Characteristics of the four groups of twin pregnancies

Group 1: TTTS with sIUGR, group 2: TTTS without sIUGR, group 3: No TTTS twin with sIUGR, group 4: No TTTS twin without sIUGR.

*p*: *p* value among the four groups of twin pregnancies generate by one-way ANOVA test or Kruskal-Wallis Test

*P*1: P value between TTTS with sIUGR (group 1) and No TTTS with sIUGR (group 3)

*P*2: P value between TTTS without sIUGR (group 2) and No TTTS without sIUGR (group 4)

*P*3: *P* value between twin pregnancies with sIUGR (groups 1 + 3) and without sIUGR (groups 2 + 4)

Data are presented as Mean ± SD except the number of preeclampsia and gestational diabetes mellitus

*one-way ANOVA test,

^&^Kruskal-Wallis Test

^※^Mann-Whitney U test,

^#^Student t test

^§^Chi-square test

**Fig. 1:**
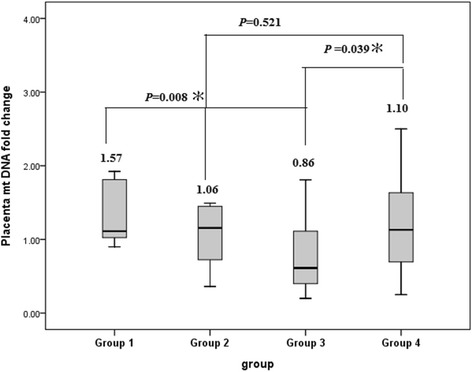
Placental mtDNA fold changes in 4 groups of monochorionic twin pregnancies. The placental mitochondrial DNA (mtDNA) fold change was defined as (placental mtDNA content of the sIUGR or smaller fetus) / (placental mtDNA content of the AGA or larger fetus). Statistics are graphically presented as the median (horizontal bar), 25 and 75 percentile (box), and ± 2 standard deviations (whiskers). Means of each group of data are also indicated in numeric figures. Grouping: Group 1: TTTS with sIUGR, Group 2: TTTS without sIUGR, Group 3: No TTTS with sIUGR, Group 4: No TTTS without sIUGR. Abbreviations: TTTS: twin-twin transfusion syndrome, sIUGR: selective intrauterine growth restriction, MC: monochorionic. *: *P* value < 0.05

All of MC twins without TTTS (group 3 and 4) were found have patent inter-twin anastomoses in placental inspection. There was no residual inter-twin anastomosis found by inspection in cases of TTTS post laser therapy. There was a significant difference in placental mtDNA fold changes (FC) among the four groups of twins (one-way ANOVA test, *p* = 0.035). Post- hoc comparison revealed that the placental mtDNA FC was significantly higher in TTTS with sIUGR (group 1) than in the no TTTS twins with sIUGR (group 3) (least significant difference, LSD test, *p* = 0.008), but the difference was not significant between group 2 and group 4 (Fig. 1). Interestingly, the no TTTS twins with sIUGR (group 3) exhibited a significantly lower number of placental mtDNA FC than the no TTTS twins without sIUGR (group 4) (LSD, *p* = 0.039). Of note, although the averaged FC in group 3 was less than 1 (0.86), the difference between placental mtDNA amount in the sIUGR and that in the AGA twins was not statistically significant (*p* = 0.228).

**Fig. 2:**
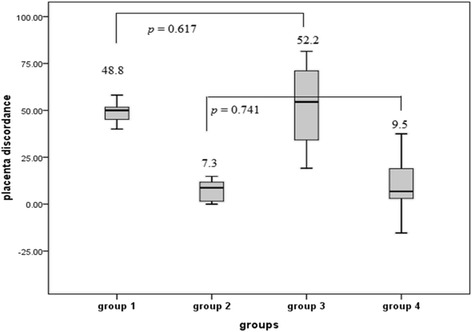
The placental discordance of four groups of monochorionic twin pregnancies. The discordance of placental shares between twins was defined as (individual placenta mass of the larger twin –individual placenta mass of the smaller twin) × 100% / (individual placenta mass of the larger twin). Statistics are graphically presented as the median (horizontal bar), 25 and 75 percentile (box), and ± 2 standard deviations (whiskers). Means of each group of data are also indicated in numeric figures. Grouping: Group 1: TTTS with sIUGR, Group 2: TTTS without sIUGR, Group 3: No TTTS with sIUGR, Group 4: No TTTS without sIUGR. Abbreviations: TTTS: twin-twin transfusion syndrome, sIUGR: selective intrauterine growth restriction, MC: monochorionic. *: *P* value < 0.05

To exclude the possibility that the difference in placental mtDNA FC between group 1 and group 3 twins was caused by discordance in the different placental shares, we also compared the placenta share discordance between groups 1 and 3 twins, and between groups 2 and 4 (Fig. 2). There were no significant differences in the placental weight discordance between groups 1 and 3 twin pregnancies which were the groups with sIUGR (*P* = 0.617) or between groups 2 and 4 twin pregnancies which were the groups without sIUGR (*P* = 0.741).

## Discussion

The fetoscopic laser therapy [20] is currently recognized as the first-line treatment for stage I to IV TTTS diagnosed before 26 weeks of gestation, resulting in better survival and neurological outcomes of fetuses. However, because laser therapy has to coagulate the placental anastomotic vessels between the two fetuses in TTTS, the decreased placental perfusion to the donor twin with IUGR may cause a higher fetal demise rate than in the TTTS donor without IUGR [21]. Furthermore, the procedure of laser therapy is not free of complications. In our series, 11.4% of treated cases experienced premature rupture of membranes (PROMs) within 3 weeks, and 2.2% of treated cases developed chorioamnionitis needing termination [13]. So in the TTTS twins where the donor has IUGR and abnormal UA Doppler, the poorer fetal outcomes should be discussed with the pregnant woman. In laser therapy for TTTS with sIUGR, it is important to preserve the blood vessels supplying to the donor twin as much as possible. In the sequential selective laser photocoagulation of communicating vessels (SQLPCV) technique, occlusion of the vascular communications is done in a particular order, resulting in a transient intra-operative net transfusion to the donor twin and facilitating a better hemodynamic equilibrium [22]. SQLPCV may be the laser strategy of choice for managing the intertwin anastomoses for TTTS with sIUGR.

After laser coagulation of the inter-twin anastomoses in the placenta of TTTS twins, the placenta became functionally dichorionic, resulting in the resolution of the polyhydramnios-oligohydramnios sequence. Group 1 twins are very similar to monozygotic, dichorionic twins with sIUGR, in which the two fetuses share identical DNA content without twin-twin vascular anastomoses since they have two separate placentas, but one fetus develops IUGR. In group 1 twins, placental mtDNA contents were 1.57-fold higher in the placental territory of the sIUGR twin than that of the larger twin (Fig. 1). Our findings were similar to previous findings in singleton pregnancies, in which placental mtDNA contents are higher in pregnancies with IUGR than in normal pregnancies [8].

Our findings that placental mtDNA fold changes were significantly higher in group 1 (those without patent vascular anastomoses) than in the group 3 twin pregnancies (those with patent vascular anastomoses) indicate that blood shunting may temper the increase of the placental mtDNA content in the sIUGR twin. MtDNA increases have been associated with hypoxia in the placentas of IUGR [8]. Our results further suggest that the rescue perfusion from the AGA- to sIUGR-fetuses determines the placental hypoxic status in the sIUGR twins between these two groups.

One potential etiology of sIUGR in MC twins is its low placental share [14]. Our results (Fig. 2) of similar placental discordance in the TTTS with sIUGR (group 1) and the no TTTS twins with sIUGR (group 3) provided evidence against the hypothesis that the different degrees of low placental share in the sIUGR twins led to different placental mtDNA contents between these two groups.

We have previously reported that the fetal / placenta weight (F/P) ratio is increased in MC twins with sIUGR [23] when rescue perfusion from the larger placental territory to the sIUGR one through vascular anastomoses works to promote growth of the sIUGR twin. By this compensatory mechanism, the sIUGR twin in each pair of shunt-patent twins (group 3) gets more perfusion than its small placental territory would usually supply, resulting in the alleviation of hypoxic stressors and a decrease of mtDNA content in the sIUGR placental territory. On the other hand, laser therapy disrupts the vascular shunt between the TTTS twins and terminates the aforementioned rescue perfusion, resulting in hypoxia and, thus, increased mtDNA contents in the placental territory of the sIUGR twin. The placental mtDNA fold change between the smaller and larger twins was not different between group 2 and 4 twin pregnancies (Fig. 1). This finding shows that, in MC twins without sIUGR but with TTTS (group 2), blocking the vascular anastomoses to treat TTTS did not cause hypoxia in the placental territory of either twin and did not increase the placental mtDNA content.

It is important to point out that the fetal blood of the sIUGR twin in MC twins contains significantly higher mtDNA [16], representing a higher state of stress in the sIUGR fetus. However, the hypoxic status of the placental compartment is quite different. In the presence of vascular anastomoses between the MC twins without TTTS (which do not require laser therapy), the presence of sIUGR (group 3) triggers a rescue shunt that effectively relieves hypoxia resulting in decreased placental mtDNA fold changes.

Limitations of this study do exist. First, the case number in the TTTS with sIUGR in this study is small (*n* = 7). After laser therapy, the survival rate of both twins in MC pregnancies complicated by TTTS in our center was around 55% (55/100), and the survival rate was further decreased to 33.3% (12/36) when the TTTS twins were complicated by sIUGR in our center [21, 24]. Therefore, it was not easy to collect the 7 cases of TTTS with sIUGR with both twins surviving after laser therapy and with intact placentas after delivery. Second, we could not exclude the possibility that the increased placental mtDNA fold changes in group 1 had been caused by the insult of polyhydramnios-oligohydramnios before laser therapy, although the demographic data between groups 1 and 3 were similar **(**Table 1, *P*1). The hypothetical detrimental effects of polyhydramnios-oligohydramnios was further weakened by the findings of similar placental mtDNA fold changes between groups 2 and 4 (Fig. 1). Third, we carefully examined every placenta with visual inspection, but did not perform dye-injection study. Therefore, residual anastomoses, especially tiny ones, cannot be completely ruled out. Lastly, rinsing the placental tissues with ice-cold PBS might not completely remove the contamination of maternal blood and thereby maternal mtDNA. However, since the comparison was done in each pair of MC twins, the effect of contamination by maternal mtDNA was equally on each twin, thus being minimized. The consideration again supports the advantage of using MC twins for sIUGR studies.

## Conclusion

In MC twin with sIUGR, the placental mtDNA fold changes between the small and large twins are different between cases with and without inter-twin anastomoses. We suspect that upon the development of sIUGR in MC twins, twin-twin vascular anastomoses in the placenta serve as a mechanism for rescue perfusion from the placental territory of the AGA twin to that of sIUGR. This compensatory mechanism alleviates the placental hypoxia in the placental territory of sIUGR twin.

## Additional file

Additional file 1: Placenta mitochondria data. Code: experiment code of patients. Quintero stage: Quintero stage of TTTS cases. Group: groups of cases (Group 1: TTTS with sIUGR, Group 2: TTTS without sIUGR, Group 3: No TTTS with sIUGR, Group 4: No TTTS without sIUGR. GAD: gestational age at delivery. Birth weight (small): birth weight of smaller or sIUGR fetus. Birth weight (large): birth weight of larger of AGA fetus. Fold change: The placenta mtDNA fold change: was defined as (placental mtDNA content of the sIUGR or smaller fetus) / (placental mtDNA content of the AGA or larger fetus). (XLSX 16 kb)

## Abbreviations

AGA: Appropriate-for-gestational-age
F/P ratio: Fetal/placenta ratio
FC: Fold changes
MC: Monochorionic
mtDNA: Mitochondrial DNA
sIUGR: Selective intrauterine growth restriction
TTTS: Twin-twin transfusion syndrome
